# Supplementary material for: In Vivo Application of CRISPR/Cas9 Revealed Implication of Foxa1 and Foxp1 in Prostate Cancer Proliferation and Epithelial Plasticity
Source: Cancers (Basel). 2022 Sep 8;14(18):4381. doi: 10.3390/cancers14184381 (PMC9496785; doi:10.3390/cancers14184381)
Supplement: Supplementary file 1 [file cancers-14-04381-s001.zip › cancers-1886823-supplementary.pdf]

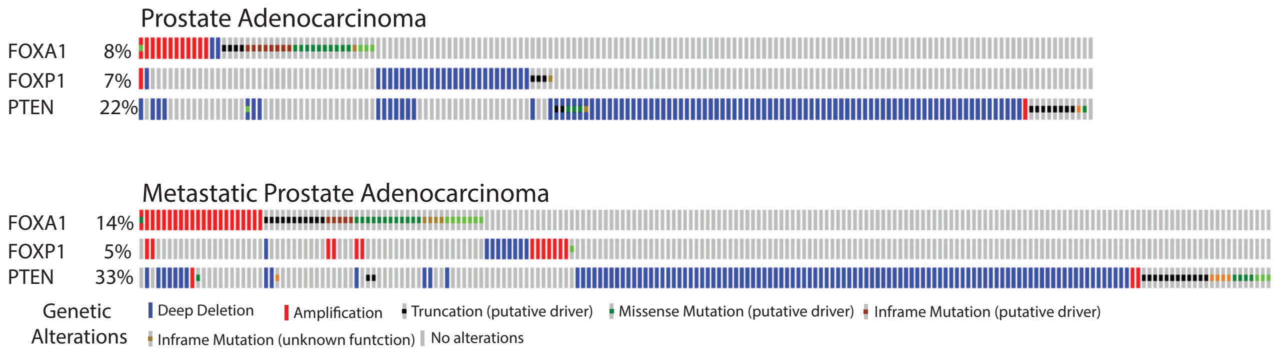

Figure S1: Loss of PTEN co-occurrence with loss of FOXP1 in primary prostate adenocarcinoma.

The mutation frequency of PTEN, FOXA1 and FOXP1 in primary PCa were assessed in a TCGA data set containing 501 samples (Firehose Legacy). The mutations burden in metastatic prostate cancer were assessed in SU2C/PCF Dream team data set containing 444 samples. PTEN co-occurrence with loss of FOXP1 in primary prostate adenocarcinoma was predicted (p=0,028). Data were generated from cBioPortal.org.

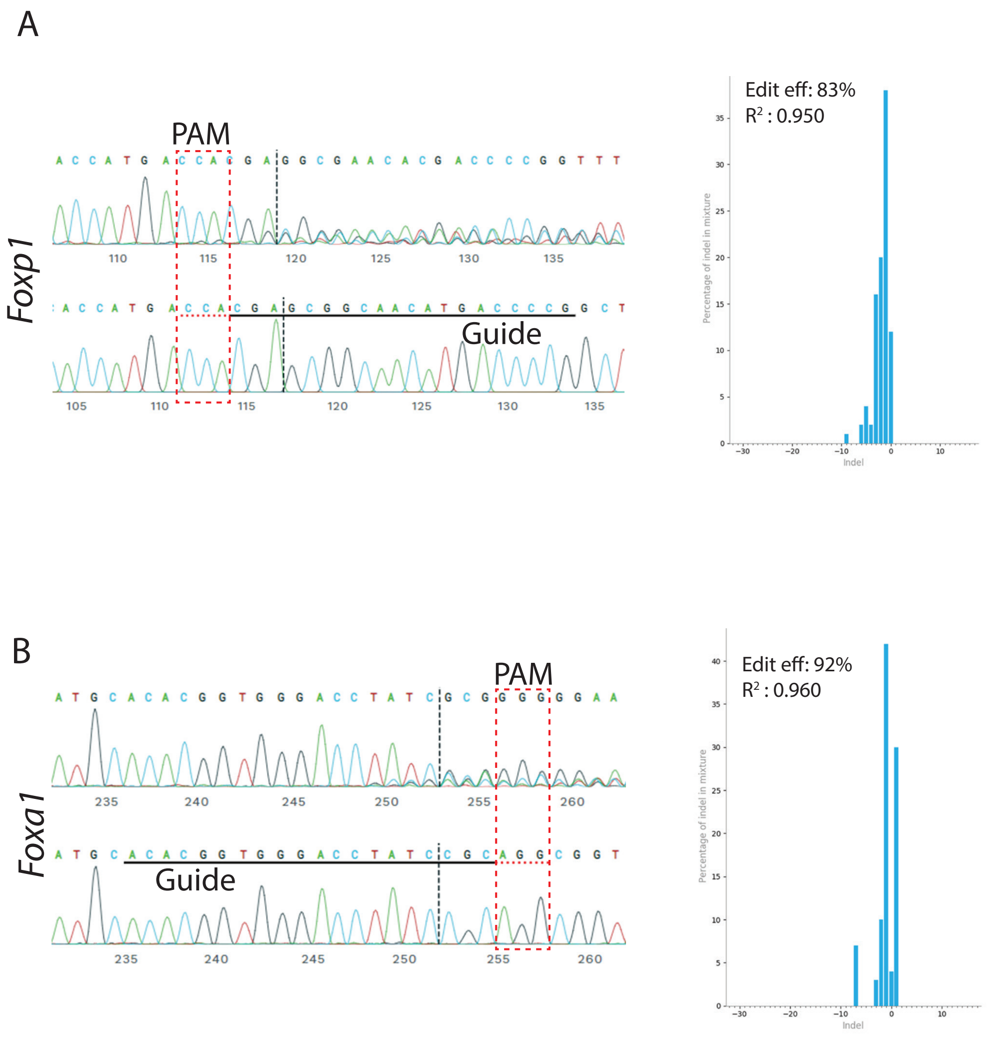

Figure S2: Guide efficiency for Foxa1 and Foxp1

MEF cells transfected with plasmid containing sgRNA's for *Foxp1* (A) and *Foxa1* (B) were assessed for Indel formation by Synthego ICE software.

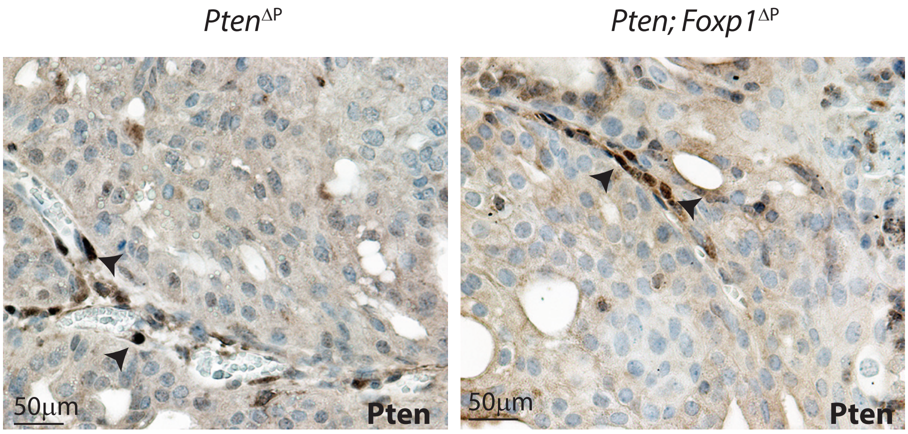

Figure S3: Loss of Pten in the prostatic tissues.

Paraffin sections from the prostates 4 months after virus delivery were stained with an antibody to Pten (brown stain). Cell marks with arrowheads shows Pten expression (n=3).

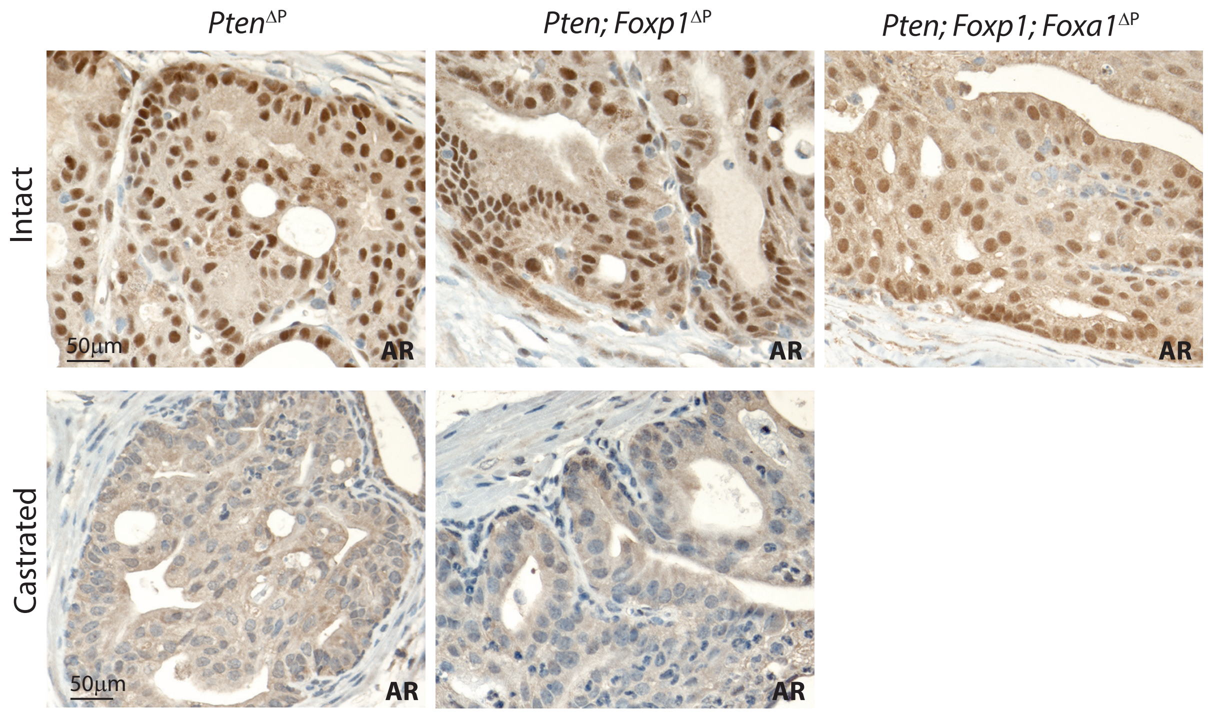

Figure S4: Normal localization of the androgen receptor in the prostatic cells.

Paraffin sections from the prostates 4 months after virus delivery were stained with an antibody to androgen receptor (AR) (brown stain) (n=3). A subset of mice was castrated one month before the experiments were terminated.

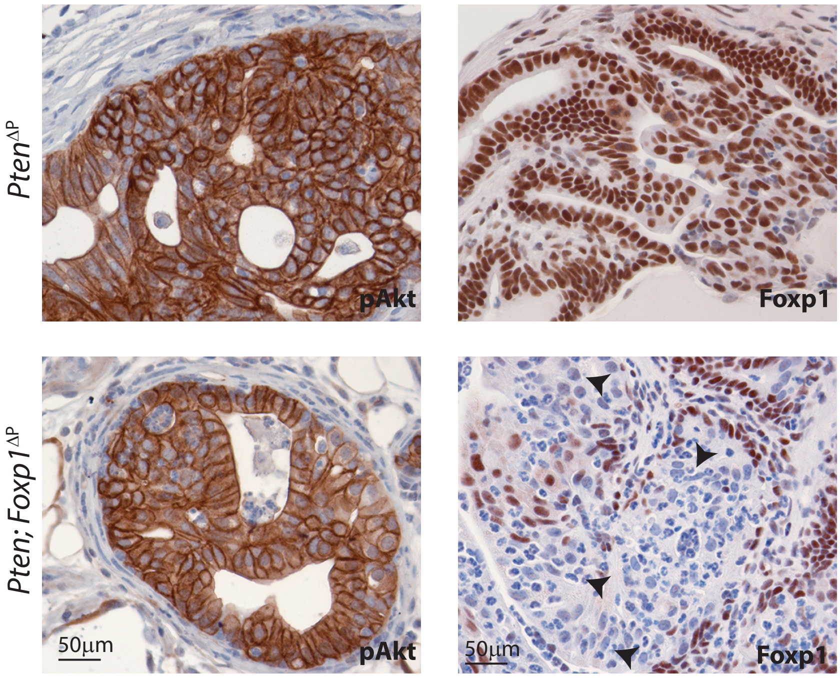

Figure S5: Castrated prostatic tissues contained loss of Foxp1 and increases p-Akt in Pten; Foxp1 deficient mice. Mice deficient for Pten or Pten; Foxp1 in the prostatic tissues underwent castration one month before samples were collected. Tissue sections were stained for p-Akt and Foxp1 (brown stain) (n=5).

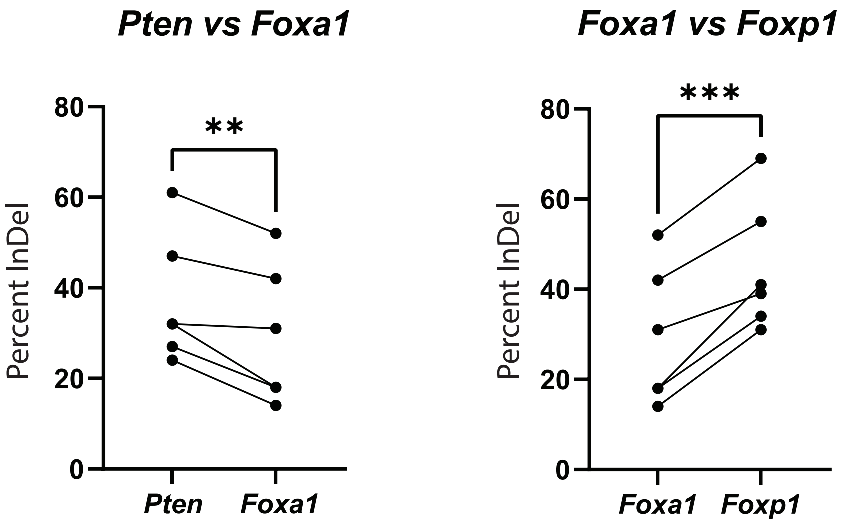

Figure S6: Decreased mutations of Foxa1 compared to Pten and Foxp1

Indel analysis for Pten, Foxa1 and Foxp1 in triple deficient samples (n=6, ** = p<0.01; *** = p<0.001).

Table S1 – oligoes

Gene	Forward primer	Reverse primer	Aim
Pten	CTCCCTGGAGTGAAGAGCAC	GTGTGCCTAGCACCTACTCC	Indel analysis
Foxa1	TCTGGGGCACACTACTAACC	GATGTAGGAGTAAGGCGGCT	Indel analysis
Foxp1	GCCAGTCTTTCCTCTGGGAC	GCATGCTTGCATACTAAGTGGT	Indel analysis
App	TCCGAGAGGTGTGCTCTGAA	CCACATCCGCCGTAAAAGAATG	qReal-Time PCR
Klk4	TTTCTGCTCGGGAGTCTTGG	CTTTCAGGTTATGCAGGCCC	qReal-Time PCR
Nkx3.1	ATGCTTAGGGTAGCGGAGC	TGCGGATTGCCTGAGTGTC	qReal-Time PCR
Tmprss2	CAGTCTGAGCACATCTGTCCT	CTCGGAGCATACTGAGGCA	qReal-Time PCR
Rpl32	GCCTCTGGTGAAGCCCAAG	TTGTTGCTCCCATAACCGATGT	qReal-Time PCR
Pten	GCTTTACAGTGAATTGCTGC	GCAGCAATTCACTGTAAAGC	Cas9 protospacer
Foxa1	CGGGGTCATGTTGCCGCTCG	CGAGCGGCAACATGACCCCG	Cas9 protospacer
Foxp1	ACACGGTGGGACCTATCCGC	GCGGATAGGTCCCACCGTGT	Cas9 protospacer
